# Allele-specific expression and high-throughput reporter assay reveal functional genetic variants associated with alcohol use disorders

**DOI:** 10.1038/s41380-019-0508-z

**Published:** 2019-09-02

**Authors:** Xi Rao, Kriti S. Thapa, Andy B. Chen, Hai Lin, Hongyu Gao, Jill L. Reiter, Katherine A. Hargreaves, Joseph Ipe, Dongbing Lai, Xiaoling Xuei, Yue Wang, Hongmei Gu, Manav Kapoor, Sean P. Farris, Jay Tischfield, Tatiana Foroud, Alison M. Goate, Todd C. Skaar, R. Dayne Mayfield, Howard J. Edenberg, Yunlong Liu

**Affiliations:** 1 Department of Medical & Molecular Genetics, Indiana University School of Medicine, Indianapolis, IN USA; 2 Department of Biochemistry & Molecular Biology, Indiana University School of Medicine, Indianapolis, IN USA; 3 Division of Clinical Pharmacology, Department of Medicine, Indiana University School of Medicine, Indianapolis, IN USA; 4 Ronald M. Loeb Center for Alzheimer’s Disease, Department of Neuroscience, Icahn School of Medicine at Mount Sinai, New York, NY USA; 5 Waggoner Center for Alcohol and Addiction Research, University of Texas at Austin, Austin, TX USA; 6 Department of Genetics, Rutgers University, Piscataway, NJ USA

**Keywords:** Neuroscience, Genetics

## Abstract

Genome-wide association studies (GWAS) of complex traits, such as alcohol use disorders (AUD), usually identify variants in non-coding regions and cannot by themselves distinguish whether the associated variants are functional or in linkage disequilibrium with the functional variants. Transcriptome studies can identify genes whose expression differs between alcoholics and controls. To test which variants associated with AUD may cause expression differences, we integrated data from deep RNA-seq and GWAS of four postmortem brain regions from 30 subjects with AUD and 30 controls to analyze allele-specific expression (ASE). We identified 88 genes with differential ASE in subjects with AUD compared to controls. Next, to test one potential mechanism contributing to the differential ASE, we analyzed single nucleotide polymorphisms (SNPs) in the 3′ untranslated regions (3′UTR) of these genes. Of the 88 genes with differential ASE, 61 genes contained 437 SNPs in the 3′UTR with at least one heterozygote among the subjects studied. Using a modified PASSPORT-seq (parallel assessment of polymorphisms in miRNA target-sites by sequencing) assay, we identified 25 SNPs that affected RNA levels in a consistent manner in two neuroblastoma cell lines, SH-SY5Y and SK-N-BE(2). Many of these SNPs are in binding sites of miRNAs and RNA-binding proteins, indicating that these SNPs are likely causal variants of AUD-associated differential ASE. In sum, we demonstrate that a combination of computational and experimental approaches provides a powerful strategy to uncover functionally relevant variants associated with the risk for AUD.

## Introduction

Alcohol use disorder (AUD) is a major public health problem [1–3]. Alcohol is a central nervous system depressant, and high levels of consumption over a long period may alter brain function to promote AUD and damage the brain, in part by altering gene expression levels [4, 5]. Understanding the molecular mechanisms by which alcohol affects the brain is important and might provide clues to the causes of AUD and ways to reverse the impact on the brain of heavy drinking.

Variations in many genes influence the risk for AUD; however, aside from functional variants in two alcohol-metabolizing enzymes, alcohol dehydrogenase and aldehyde dehydrogenase, each individual variant has only a small effect [1, 3, 6, 7]. In addition to genetic differences, environmental factors, and interactions among the variants also affect AUD risk [1, 3, 6]. Genome-wide association studies (GWAS) identify regions in the genome that affect risk for complex diseases [8], but to date, only a few AUD-associated loci have been unambiguously identified [7]. Most identified regions contain many variants that are inherited together (linkage disequilibrium), and identifying the causal variant amongst all the associated ones is a major challenge.

A powerful method to study the effects of genetic variants on gene regulation is to examine allele-specific expression (ASE). ASE measures the difference in expression between the alternative alleles and is regulated by *cis*-acting DNA elements, since both alleles are exposed to the same *trans*-acting environment in the cell. ASE analysis can be used as a marker to identify strong candidate genes with AUD-associated differential expression. ASE in genes could be influenced by several mechanisms; one is by functional variants in the 3′ untranslated regions (3′UTRs). Single nucleotide polymorphisms (SNPs) within 3′UTRs can modulate gene expression as target sites for micro-RNAs (miRNAs) or RNA-binding proteins (RBPs). A high-throughput reporter assay offers a powerful tool to screen hundreds of genomic variants associated with a specific phenotype to determine which variants affect gene regulation. The combination of ASE analysis and a high-throughput assay provides a unique strategy to uncover candidate genes contributing to AUD risk and to identify functionally relevant variants in those genes. Together, these complementary approaches provide a mechanism of action and thereby advance our understanding of the biology of AUD.

Here, we integrated deep RNA-seq and SNP genotyping data from four different brain regions of 30 subjects with AUD and 30 social/non-drinking control subjects to detect genes whose ASE differs. The brain regions were: (i) the basolateral nucleus of the amygdala (BLA), which plays crucial roles in stimulus value coding and alcohol withdrawal-induced increase in anxiety [9]; (ii) the central nucleus of amygdala (CE), which mediates alcohol-related behaviors and alcohol dependency [10]; (iii) the nucleus accumbens (NAC), in which alcohol promotes dopamine release [11]; and (iv) the superior frontal cortex (SFC), which is implicated in cognitive control and experiences neuronal cell loss after long-term alcohol abuse [12]. We then used a high-throughput reporter assay called PASSPORT-seq (parallel assessment of polymorphisms in miRNA target-sites by sequencing) [13] to systematically screen all the SNPs in the 3′UTR regions of the genes that demonstrated differential ASE between AUD and control subjects, in any of the four brain regions, to determine which variants altered RNA levels in cells of neural origin. We identified 25 functional SNPs that altered gene expression in the same direction in both SH-SY5Y and SK-N-BE(2) cell lines. Many of these SNPs are located on the binding sites of miRNAs and RBPs.

## Materials and methods

### Human brain tissues

The human brain tissues were obtained postmortem from four brain regions of 60 individuals (30 subjects with AUD and 30 social/non-drinkers) from the New South Wales Brain Tissue Resource Center (NSWBTRC) at the University of Sydney. Alcohol-dependent diagnoses of the 30 AUD subjects met the American Psychiatric Association DSM-IV criteria [14], and were confirmed by physician interviews, review of hospital medical records, questionnaires to next-of-kin, and from pathology, radiology, and neuropsychology reports. The control group consisted of 30 social/non-drinker samples, which were matched with the 30 AUD subjects based on age, sex, postmortem interval, pH of tissue, and cause of death. Additional selection criteria included >18 years of age, no head injury at time of death, lack of developmental disorder, no recent cerebral stroke, no history of other psychiatric or neurological disorders, no history of intravenous or polydrug abuse, negative screen for AIDS and hepatitis B/C, and postmortem interval within 48 h.

### RNA-seq and genotyping

RNA was extracted from the brain tissues using the Qiagen RNeasy kit (Qiagen, Germantown, MD, USA). The RNA-seq samples were prepared using the TruSeq RNA Library Pre Kit v2 (Illumina, Inc., San Diego, CA, USA) and sequenced on the Illumina HiSeq 2000. Paired-end libraries with an average insert size of 180 bp were obtained. Raw reads were aligned to human genome 19 (hg19) using STAR aligner version 2.5.3.a [15]. FastQC (https://www.bioinformatics.babraham.ac.uk/projects/fastqc/) was used to evaluate RNA sequence quality. The RNA-seq data is available through the NCBI BioProject database (BLA: PRJNA551909, CE: PRJNA551908, NAC: PRJNA551775, SFC: PRJNA530758).

DNA was obtained from the same brain tissues. The Axiom Biobank Genotyping Array (Thermo Fisher Scientific, Waltham, MA, USA) was used for genotyping. Monomorphic variants, variants with call rate ≤0.98 or Hardy–Weinberg equilibrium *p* < 10^−5^, and samples with call rate <0.90 were removed using PLINK [16]. Phasing was done using SHAPEIT2 [17]. IMPUTE2 [18] was used for imputation using the 1000 Genomes Phase 1 integrated panel (excluding singleton variants) as the reference. Variants with imputation score ≥0.8 and estimated minor allele frequency (MAF) ≥0.5 were included in the analysis.

### ASE analysis

GATK ASEReadCounter was used to obtain reference and alternative allele counts at the exonic SNPs [19]. We only analyzed SNPs that were heterozygous in at least five control and five AUD groups, and that also had more than 10 reads, to ensure that samples with sufficient reliable read counts were used for analysis.

We used a generalized linear mixed effect model (GLMM) to model the number of RNA-seq reads for each allele based on its allelic type (reference or alternative allele), study group (AUD or social/non-drinkers), and the interaction terms between the two variables. A random variable was used to account for the reads from the two alleles within the same individual. To adjust for the over-dispersion effects of the RNA-seq reads, a negative binomial distribution was used in the GLMM model.

$${\mathrm{{log}}}(\mu ) = \beta _0 + \beta _1X_1 + \beta _2X_2 + \beta _{12}X_1X_2 + bX_{\mathrm{{S}}}$$

where *μ* is the expected number of sequencing reads for one allele (reference or alternative) in one specific subject, *X*_1_ is the allele type (0: reference allele and 1: alternative allele), *X*_2_ is the subject study group (0: control group and 1: AUD group), and *X*_S_ is the subject ID. In this model, *β*_0_, *β*_1_, *β*_2_, and *β*_12_ are coefficients of fixed effects, while *b* is the coefficient for random effect that models the differences between subjects. Our null hypothesis (H_0_) is: *β*_12_ = 0. Rejecting the null hypothesis indicates that the allelic imbalance differs between the AUD and control groups. False discovery rate (FDR) was calculated using the Benjamini–Hochberg procedure [20]. Ingenuity pathway analysis (IPA) (Qiagen) was used for deriving the pathways of the genes with AUD-associated allelic differences.

### Screening for functional SNPs in the 3′UTR

**Fig. 1:**
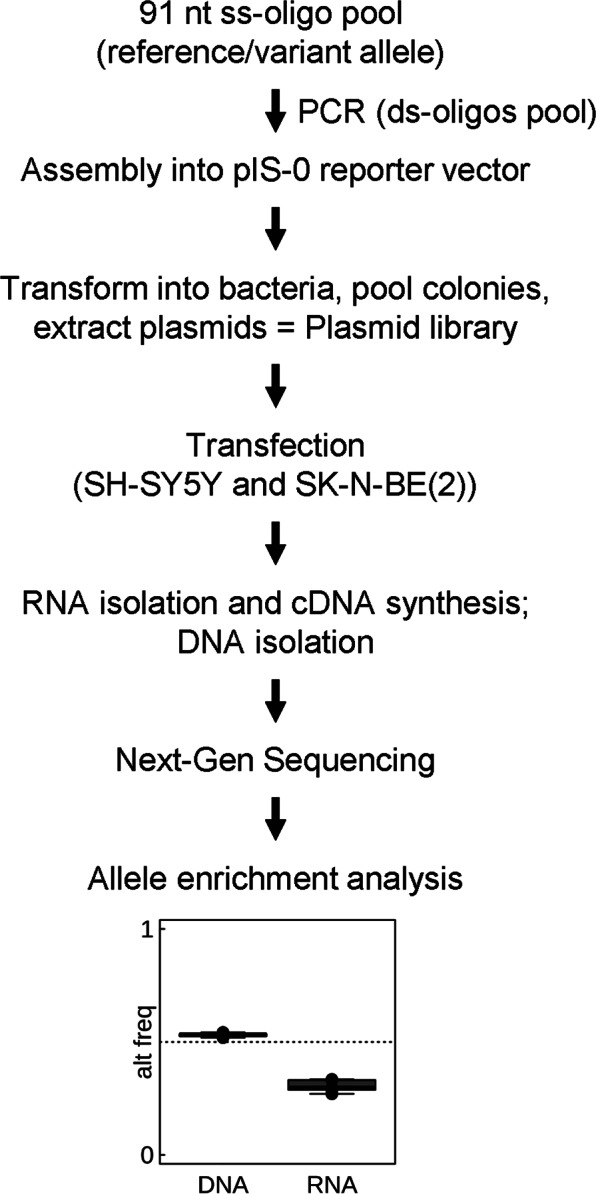
Schematic representation of the PASSPORT-seq assay. A pool of oligonucleotides flanking both alleles of 437 SNPs were cloned in parallel into the 3′UTR of the luciferase gene in pIS-0 vector. Colonies were pooled and DNA purified. The resulting plasmid library was transiently transfected into two neuroblastoma cells lines, SH-SY5Y and SK-N-BE(2). The cDNAs were synthesized from the total RNA. The target sequences were amplified from the cDNAs and the plasmid DNA extracted from the transfected cells using two-step-PCR with unique barcodes for each cell line and each biological replicate. Following next-generation sequencing, the reads were aligned to the reference library consisting of all the test sequences and ASE was measured for each SNPs. ss single-stranded, ds double-stranded

The PASSPORT-seq assay was conducted as previously described [13] with some modifications described in Supplementary Materials. Briefly, the procedure includes the following steps: oligonucleotide synthesis, plasmid library construction, transfection, DNA/RNA extraction, and sequencing. The oligonucleotide pool was synthesized commercially (Oligomix^®^, LC Sciences, Houston, TX, USA), and included 874 DNA oligos to target both alleles of 437 SNPs in the 3′UTR of genes that showed differences in allelic ratio between control and AUD groups. These were cloned in parallel into the 3′UTR of the luciferase gene in the pIS-0 vector (12178, Addgene, Cambridge, MA, USA). The resulting plasmid pool was purified from transformed DH5alpha bacteria and transfected into two human neuroblastoma cell lines, SH-SY5Y and SK-N-BE(2). Each transfection was repeated six independent times. Both plasmid DNA and cellular RNA were extracted from the cells 42 h post-transfection. Plasmid DNA and cDNA sequences containing the cloned SNPs were amplified using PCR primers that also incorporated sample barcodes, unique molecular indices (UMI), and Illumina-sequencing adapters. The resulting PCR products were sequenced using one lane of Illumina HiSeq 4000 using a 75 bp paired-end protocol. The detailed experimental protocol is provided in the Supplementary Materials. A schematic of the overall procedure is shown in Fig. 1.

FASTQ files were demultiplexed using cutadapt [21] based upon barcodes identifying DNA source and replicate number, and the barcodes and adapter sequences were trimmed from each read. The UMI was then trimmed and stored in the read name using umi_tools [22]. Using bwa-mem [23], remaining reads were mapped to the reference sequences defined by the reference and alternative sequence for each SNP of interest. Finally, umi_tools was used to count the number of UMI-unique reads for each sequence.

To identify the functional 3′UTR SNPs, i.e. those whose ratio between reference and alternative allele was significantly different between the vector-expressed RNA and the plasmid DNA extracted from the same transfected cells, a generalized linear model was used. Similar to ASE analysis, a negative binomial distribution was used to account for the over-dispersion effects on the UMI count data.

### Annotation of the functional 3′UTR SNPs

For each SNP, expression quantitative trait loci (eQTLs), RBP-binding sites, and miRNA target sites were annotated. The eQTL information was retrieved from the Genotype-Tissue Expression (GTEx) database (version V7; accessed December 17, 2018) [24] to find SNPs that are eQTLs for the same gene, and also to determine whether miRNAs were expressed in brain. The UCSC Genome Browser database [25] was used to annotate whether a specific SNP was located in the binding site of an RBP using the ENCODE RNA-Binding Protein track [26]. The Polymirts database [27] was used to predict the potential miRNAs whose binding can be altered by a candidate SNP, where the binding of an miRNA on a specific target sequence was evaluated by TargetScan [28].

### Ethanol treatment of cells

To evaluate the potential effect of alcohol on the function of the 3′UTR SNPs, untransfected SK-N-BE(2) cells were grown to confluence in a 9.5 cm^2^ CellBIND six-well dish (Corning, Corning, NY, USA) and then treated with physiological concentrations of ethanol (10 or 20 mM) or left untreated. Following 24 and 42 h ethanol treatment, cells were harvested for RNA isolation, library preparation, and RNA sequencing. For the 42 h treatment condition, media was replaced with fresh media with or without ethanol in both ethanol treated and control cells, respectively, at 24 h. Four independent biological replicates were conducted for each condition. The mRNA was extracted from the cells using the Qiagen RNeasy kit (Qiagen). The RNA-seq samples were prepared using the TruSeq RNA Library Pre Kit v2 (Illumina) and sequenced on the Illumina HiSeq 4000 using 2 × 75 bp paired-end configuration. A GLMM model was used to identify the variants whose allelic frequencies were altered by ethanol treatment (see Supplementary Materials). The RNA-seq data is available through Gene Expression Omnibus (GEO) database (accession number: GSE131470).

## Results

### AUD-associated differential ASE

We performed deep RNA-seq experiments (>100 million reads per sample) from each of four brain regions (BLA, CE, NAC, SFC) from 60 individuals, 30 with and 30 without AUD (240 total samples). In addition, we obtained genotypes for all 60 subjects. After aligning RNA-seq reads to the human genome and imputing the SNP array genotyping to ~10 million SNPs, we retrieved allele counts from the RNA-seq data for all SNPs for which at least one sample was heterozygous, for a total of 250,007 SNPs. We focused on the ~17,000–24,000 SNPs in each of the four brain regions that had more than 10 reads in at least five heterozygous samples in both the AUD and control groups. The overall strategy is summarized in Supplementary Fig. 1 and Supplementary Table 1.

To examine whether there were allele-specific differences that varied between subjects with AUD and controls, a GLMM was implemented; the coefficient of the interaction between allele and experimental group, *β*_12_, estimates the log2 ratio of the fraction of alternative allele reads between the control and AUD samples, and was used to evaluate whether the ASE at a specific locus was significantly different between AUD and control groups.

**Fig. 2:**
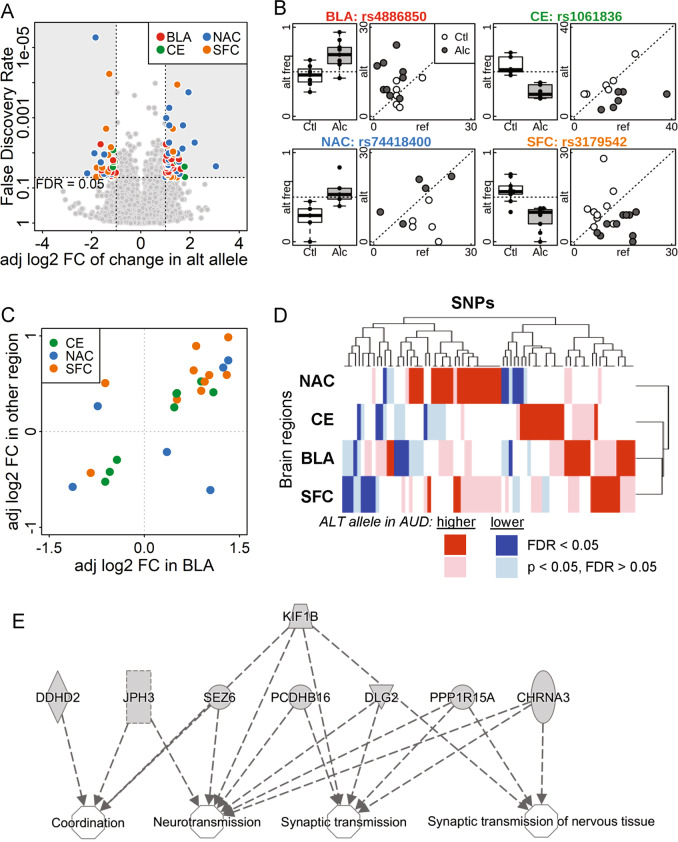
Allele-specific expression in the postmortem brain samples from subjects with and without AUD. **a** Volcano plot comparing the adjusted log2 fold change (adj log2 FC) and false discovery rate (FDR) of the percentage of alternative alleles between AUD subjects and controls. SNPs with FDR < 0.05 and adjusted log2(fold change) >1 or <−1 were color-coded by brain region. BLA basolateral nucleus of the amygdala, CE central nucleus of the amygdala, NAC nucleus accumbens, and SFC superior frontal cortex; alt alternative. **b** Alternative allele frequency box plot and a scatter-plot of the number of reference and alternative reads for one significant SNP from each brain region (symbols noted above). alt freq alternative allele frequency; Ctl = social/non-drinking control subjects; Alc = AUD subjects; ref = reference. **c** Consistency in the adj log2 FC in different brain regions. SNPs with FDR < 0.05 in BLA and *p* < 0.05 in another brain region are plotted by adjusted fold change, color-coded by the other brain region. Of the 24 SNPs, 20 had consistent fold change direction in the two brain regions. **d** Heatmap of adjusted fold change of SNPs with FDR < 0.05 in at least one brain region shows consistency in fold change among all four brain regions. Dark and light colors indicate genome-wide significant (FDR < 0.05), or borderline significant (FDR > 0.05 but *p* < 0.05), respectively. Red and blue indicate increased and decreased percentage of alternative allele in the AUD brain comparing to control group. **e** Ingenuity pathway analysis results of genes enriched in nervous system development and function

We identified 88 SNPs with ASE in at least one brain region that significantly differed between subjects with and without AUD (Fig. 2a) at an FDR < 0.05 and |*β*_12_| > 1. Among these SNPs, 58 showed an increased fraction of the alternative allele in AUD samples, while 32 showed a decreased fraction. There were 26 SNPs in the BLA, 9 in the CE, 31 in the NAC, and 22 in the SFC. Examples of SNPs with significant differential ASE in each brain region are shown in Fig. 2b. An annotated list of these 88 SNPs, including their loci, allele frequencies, gene, and functional location, is provided in Supplementary Table 3. In addition, we developed an R-shiny-based interactive website (https://yunlongliulab.shinyapps.io/aud_ase/) to provide a direct visualization of the ASE findings. This data portal exhibits the RNA-seq read counts on both reference and alternative alleles at each significant ASE locus for every subject.

Among the SNPs with differential ASE, many showed the same direction of effect (*p* < 0.05) in more than one brain region (although the FDR might have been >0.05 in another region). For example, among the 92 SNPs with ASE in BLA that had FDR < 0.05 (no requirement for *β*_12_), 24 also showed changes in other brain regions (*p* < 0.05), of which 20 had consistent *β*_12_ direction in at least one of the other regions (Fig. 2c). Similar consistency was observed in significant SNPs (FDR < 0.05) in CE, NAC, and SFC (Supplementary Fig. 2). As shown in the heatmap (Fig. 2d), among the SNPs with FDR < 0.05 in at least one brain region, a similar trend of AUD-associated allelic bias was also observed in at least one other region.

The genes containing the 88 ASE SNPs were analyzed using IPA; 17 were involved in nervous system development and function or neurological disease (enrichment *p*-value ranged from 8.8E−04 to 3.7E−02), including neurotransmission and coordination (Fig. 2e), movement disorders and neuromuscular disease.

### Functional SNPs in 3′UTRs

To understand a potential mechanism of action responsible for the ASE, we tested 3′UTR SNPs of the genes with ASE to determine whether they affected gene regulation. Because both heterozygous alleles exist in the same cell and are exposed to the same environment, ASE differences arise due to regulation in *cis*. Among the genes with AUD-associated differences in ASE in at least one of the four brain regions, we identified 565 SNPs in the 3′UTR regions; 437 SNPs in 61 genes had at least one heterozygote in either the AUD or control group (Supplementary Table 3). We adapted a high-throughput reporter assay, PASSPORT-seq [13], to identify 3′UTR variants that affected gene expression (RNA levels) in two human neuroblastoma cell lines, SH-SY5Y and SK-N-BE(2) (Supplementary Table 4). We detected expression of the reference and alternative alleles in both SH-SY5Y and SK-N-BE(2) cells in 362 (82.8%) of the 437 SNPs screened. UMIs were counted to quantify the expression of the reference and alternative alleles for each SNP, and a generalized linear model was applied to identify the variants that showed ASE.

**Fig. 3:**
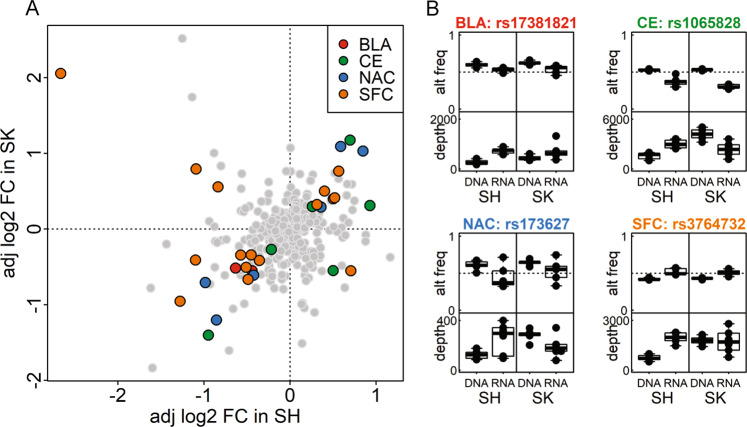
PASSPORT-seq results in SH-SY5Y and SK-N-BE(2) cell lines. **a** Plot of the adjusted log2(fold change) of alternative allele frequency between RNA and DNA in SH-SY5Y [SH] and SK-N-BE(2) [SK] cell lines in the four brain regions. SNPs with FDR < 0.05 in both cell lines were color-coded by the brain region. **b** Alternative allele frequency and read depth for significant SNPs derived from each brain region

As shown in Fig. 3a, 53 of the detected SNPs showed significant differences in ASE (FDR < 0.05) in SH-SY5Y cells and 130 in SK-N-BE(2) cells. Thirty of these SNPs showed significant ASE in both cell lines, with a consistent direction of allele imbalance in 25 SNPs: 2 from BLA, 5 from CE, 6 from NAC, and 12 from SFC (Fig. 3a). The alternative allele frequencies and sequencing depths of four representative SNPs (one from each region) are shown in Fig. 3b. Significant variants in all brain regions are shown in Supplementary Fig. 3.

We annotated the 25 variants that showed ASE in the same direction in both SH-SY5Y and SK-N-BE(2) cells (Table 1). Eighteen were found to be in eQTL regions of their own genes, based on the GTEx database [22], which provides additional evidence that the identified genes are, at least in part, regulated by the *cis*-acting variants. In addition, 14 SNPs coincided with the binding site of one or more RBPs. Interestingly, poly(A)-binding protein cytoplasmic 1 (*PABPC1*) was associated with the location of 11 SNPs, while ELAV-like RBP (*ELAVL1*) was associated with 7. TargetScan matching of miRNA seed sites at each variant location using PolymiRTS [27] found 10 SNPs that disrupt or introduce a miRNA-binding site. Four of these SNPs interfere with the seed sites of 13 miRNAs that are known to be expressed in brains (Table 1).

**Table 1:** Annotations for SNPs that showed significant and consistent impacts in both SH-SY5Y and SK-N-BE(2) cell lines by PASSPORT-seq

Region	Gene	SNP	MAF (%)	eQTL	RBP binding	Potential miRNA target
BLA	*PEAK1*	rs17381821	18	Yes		miR-3913-3p^a^
BLA	*PNP*	rs1079375	32	Yes		
CE	*HELQ*	rs4693089	34	Yes	PABPC1, ELAVL1	
CE	*KIF1B*	rs1065828	33	Yes	IGF2BP1	
CE	*KIF1B*	rs1536262	45	Yes	IGF2BP1	
CE	*PACRGL*	rs6812257	31	Yes	ELAVL1, PABPC1	
CE	*ZNF625*	rs59816741	1	No		
NAC	*LYPD5*	rs4525602	3	No		
NAC	*PCDHB16*	rs2338530	10	Yes	PABPC1	
NAC	*PRLR*	rs173627	24	No	PABPC1	
NAC	*SLC16A4*	rs11120	18	Yes	ELAVL1, PABPC1	miR-200b-3p^b^, miR-200c-3p^a^, miR-374c-5p^a^, **miR-429**^c^, miR-4330^b^, miR-510-5p^a^, miR-512-5p^b^, miR-655-3p^b^, miR-8084^a^, **miR-3942-3p**^c^, **miR-506-5p**^c^, miR-5100^b^, miR-6074^a^, miR-889-3p^c^, miR-892c-5p^a^
NAC	*SMPD4*	rs10909567	38	Yes	IGF2BP1	
NAC	*SYT9*	rs10732510	48	No		miR-3925-3p^b^, **miR-136-5p**^c^, miR-4695-3p^a^, miR-665^a^, **miR-766-3p**^c^
SFC	*CA13*	rs4617148	45	Yes	ELAVL1, PABPC1, IGF2BP1	**miR-3125**^c^, **miR-3916**^c^, miR-6859-5p^a^, miR-6866-5p^a^, **miR-877-5p**^c^, **miR-4496**^c^
SFC	*CA13*	rs4740047	42	Yes	ELAVL1, PABPC1, IGF2BP1	
SFC	*FKTN*	rs2010861	13	Yes	ELAVL1, PABPC1	miR-6840-3p^a^
SFC	*LRPAP1*	rs11723071	20	Yes		
SFC	*LRPAP1*	rs11729369	25	Yes		
SFC	*LRPAP1*	rs13325	13	No		
SFC	*LRPAP1*	rs140653273	1	No		
SFC	*MAVS*	rs6515831	31	Yes		miR-4289^a^
SFC	*PODN*	rs899974	19	No		
SFC	*PPARA*	rs10154348	7	Yes	ELAVL1, PABPC1, CELF1	miR-7155-5p^a^
SFC	*SNX18*	rs2565014	35	Yes	PABPC1	miR-4655-3p^a^ miR-7848-3p^a^
SFC	*UQCC1*	rs3764732	14	No	PABPC1	miR-3922-5p^a^, miR-513c-5p^b^, **miR-514b-5p**^c^, **miR-330-3p**^c^, miR-371a-5p^a^, **miR-371b-5p**^c^, miR-372-5p^a^, miR-373-5p^b^, **miR-616-5p**^c^, miR-7109-3p^a^

Brain regions: *BLA* basolateral nucleus of the amygdala, *CE* central nucleus of amygdala, *NAC* nucleus accumbens, *SFC* superior frontal cortex. *MAF* minor allele frequency, *eQTL* whether the SNP had a significant eQTL for its gene in GTEx, *RBP* RNA-binding proteins whose binding sites overlapped the SNP, and miRNAs whose binding potential was changed by the alternative allele

^a^miRNA expression unknown

^b^miRNA not expressed in brain

^c^miRNA expressed in brain

### Ethanol treatment changes the ASE in SNPs of interest

**Fig. 4:**
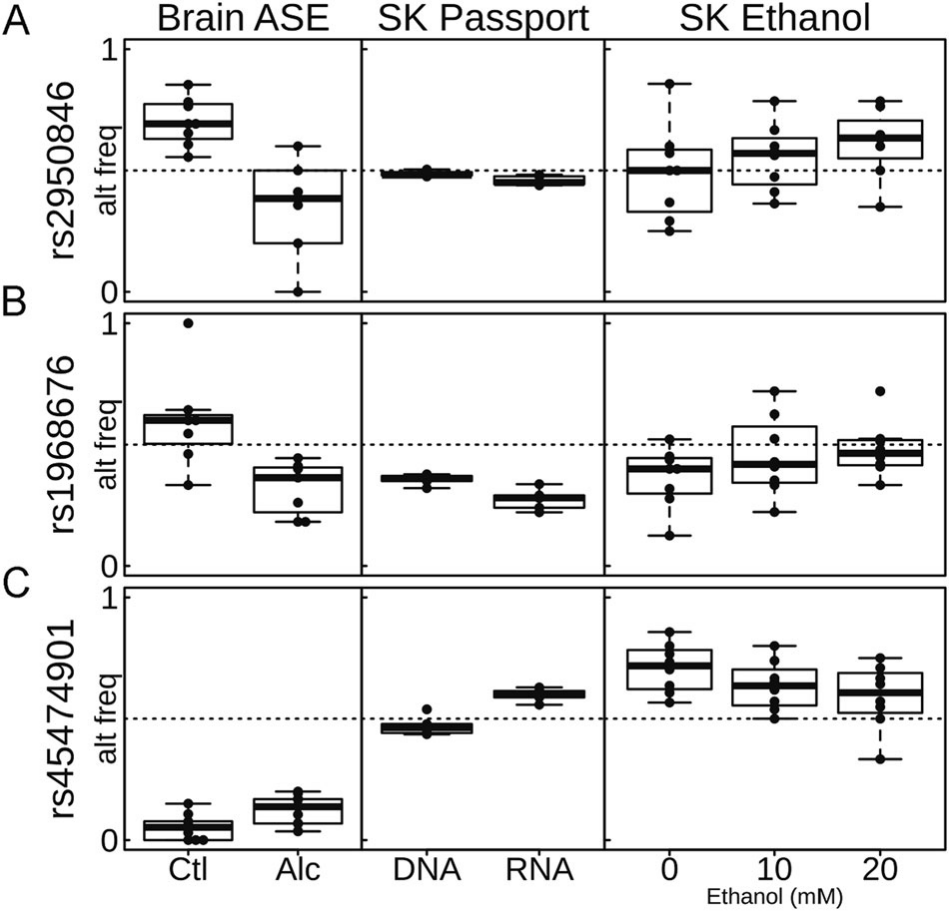
ASE, PASSPORT-seq, and ethanol treatment results for selected SNPs. Three SNPs (rs2950846, rs1968676, rs45474901) that had alternative allele frequency significantly different in PASSPORT-seq results (FDR < 0.05) and significantly different across 0, 10, and 20 mM ethanol treatment dosages in SK-N-BE(2) cells (*p* < 0.05) were identified. The differences in the alternative allele frequency in the brains of heterozygous social/non-drinkers (Ctl) and AUD subjects (Alc) are also shown. SK = SK-N-BE(2) cells

To evaluate the impact of alcohol treatment on the ASE of endogenous SNPs, we examined their expression in SK-N-BE(2) cells before and after treatment with two concentrations of ethanol, 10 and 20 mM. Among the 130 PASSPORT-seq SNPs showing ASE in SK-N-BE(2) cells, 17 had an average read depth per sample >15 and had at least 10% of their reads supporting the minor allele in either the ethanol-treated or untreated SK-N-BE(2) cells. Of these, we identified six SNPs whose ratios between reference and alternative alleles were significantly altered by alcohol treatment in a dose-responsive manner: rs45474901 (*p* = 0.014), rs2950846 (*p* = 0.017), rs45522239 (*p* = 0.022), rs45548238 (*p* = 0.027), rs1968676 (*p* = 0.029), and rs2338530 (*p* = 0.041). Three of these SNPs are located in the 3′UTR of *TMEM25* (*transmembrane protein 25*), two are within *PCDHB16* (*protocadherin beta 16*), and one is in *SMPD4* (*sphingomyelin phosphodiesterase 4*). The SNP with the smallest *p-value* in each gene is shown in Fig. 4 (the other three SNPs are shown in Supplementary Fig. 4). For five of these six SNPs, the effects of acute ethanol treatment were in the opposite direction of that seen in the brains of subjects with AUD. For example, the variant rs45474901 allele in the 3′UTR of *TMEM25* exhibited higher expression in the PASSPORT-seq assay and in SFC of heterozygous subjects with AUD, but lower expression after ethanol exposure in SK-N-BE(2) cells. Conversely, for rs1968676, a SNP in *SMPD4*, and rs2950846, a SNP in *PCDHB16*, the variant alleles were expressed at lower levels in AUD subjects and in PASSPORT-seq but at higher levels after ethanol treatment.

## Discussion

In this study, we used two tightly coupled approaches, ASE analysis together with a high-throughput reporter assay, as an innovative strategy to discover the roles of *cis*-acting variants on gene regulation in AUD. We identified 88 genes whose ASE differed between AUD and control subjects in at least one of four brain regions; many of the differences were consistent in direction across brain regions. Pathway analysis showed enrichment of these genes in pathways related to neurological disorders and neurodegenerative diseases. High-throughput screening in SH-SY5Y and SK-N-BE(2) cell lines identified 53 and 130 SNPs in the 3′UTR of these genes, respectively, that showed significant ASE, among which 25 SNPs lead to ASE in the same direction in both cell lines.

Differences in ASE between subjects with and without AUD could be preexisting or the result of decades of excessive alcohol consumption. The functional SNPs, however, were detected by PASSPORT-seq in cultured cells in the absence of ethanol and therefore likely affect expression in the brain even in absence of ethanol exposure. Interestingly, the ASE of six SNPs in SK-N-BE(2) cells was altered by pharmacologically relevant concentrations of ethanol (10–20 mM); the direction of the acute alcohol response was opposite to the direction in the brain regions. Similar trends have been observed by others, e.g. acute alcohol exposure favors an anti-inflammatory response and chronic alcohol consumption favors proinflammatory cytokine release [29, 30]. The SNPs with in vitro ethanol-induced changes in allelic ratio are located in the 3′UTR of the three genes, *PCDHB16*, *TMEM25*, and *SMPD4*. *PCDHB16* is a potential calcium-dependent cell-adhesion protein that may be involved in the establishment and maintenance of specific neuronal connections in the brain. The expression of clustered protocadherins has been shown to be strongly linked with selection for ethanol preference in mice [31, 32]. In addition, the expression of *PCDHB16* is increased in the mouse NAC (the same region we found ASE in this study) in response to cocaine [33]. *TMEM25* encodes a transmembrane protein that is expressed in multiple brain regions, including the cerebellar cortex and hippocampus, as well as in neuroblastoma and brain tumors, and may be involved in the promotion of axon growth and the regulation of cell migration [34, 35]. *SMPD4* encodes an enzyme sphingomyelinase that catalyzes the hydrolysis of sphingomyelin into phosphorylcholine and ceramide and is important in maintaining sphingolipid metabolism. It is one of the neutral sphingomyelinases whose activity is increased in HepG2 cells [36] and in astrocytes [37] in response to alcohol. Neutral sphingomyelinases are highly active in brain regions [38] and are suggested to play an important role in neurological disorders [39, 40]. For example, in brain injury, the activity of neutral sphingomyelinase is induced and the ceramide level accumulates in astrocytes after cerebral ischemia [39]. Another study showed that the activity of neutral ceramidase (an enzyme that cleaves fatty acid from ceramide) was increased in Alzheimer’s disease brain fractions [41]. In addition, treating neuronal cultures with amyloid beta peptide has been shown to elevate neutral sphingomyelinase and ceramide activities [41, 42].

ASE analysis identifies genes whose expression levels are influenced by the *cis*-acting elements and complements eQTL analysis, but with higher statistical power, because the expression signals of the two alleles in the same sample serve as internal controls for each other. Despite these advantages, ASE analysis has several challenges. First, it can only address heterozygous loci. Second, it is only powered for genomic loci with at least modest sequencing coverage (we used at least 10 reads); thus, the requirement for RNA-sequencing depth is high. In our study, the average depth for each sample was ~100 million reads. A detailed discussion about the relationship between read depth, number of samples, and statistical power for identifying ASE differences between two conditions is provided in the Supplementary Materials. Third, the ASE changes we identified could have existed prior to the excessive alcohol consumption characteristic of AUD or could have resulted from it; they may not reflect etiology of AUD. However, our approach could have broad importance as a follow-up to well-powered genetic studies by identifying variants that have functional effects on gene expression within the usually broad loci identified in such studies. Finally, ASE analysis from RNA-seq data is subject to technical challenges due to the differences in our ability to align sequence reads to two alleles. Most alignment algorithms more readily align the sequencing reads with the reference sequence, which can lead to depressed expression signals for the alternative allele. Fortunately, this potential bias can be avoided by comparing the allelic imbalances between two experimental conditions, since the biases due to alignment algorithms will be the same in both. Thus, we focused our analysis on the differences in allelic imbalance between subjects with and without AUD. This strategy also allowed us to focus our analysis on the genetic variants that were associated with AUD.

It should be noted that the heterozygous SNPs used to identify ASE are markers and not necessarily causal. Therefore, screening multiple variant loci in regulatory regions of those genes is a logical next step. Therefore, we examined the 3′UTR of the genes identified in ASE to test one potential mechanism of gene regulation. We used a high-throughput reporter assay, PASSPORT-seq [13], to screen isolated 3′UTR variants to detect those that lead to gene expression changes. We modified the original protocol to improve the quality of the data. First, we introduced UMIs during the reverse transcription of the mRNA and in the first PCR of the plasmid DNA used as a control to overcome potential PCR biases during the library construction. Second, we added staggered sequences to the PCR primers to reduce problems associated with low sequencing complexity at the beginning of the reads (see Supplementary Materials). A limitation is that we only screened 3′UTR variants; variants in other regulatory regions, such as enhancers and promoters, also play important roles in cis-acting regulation.

In summary, this study identified a subset of genes that show differential ASE between subjects with and without AUD; the differences might preexist and affect the risk for AUD or might result from alcohol-induced neurological damage. Within those genes, we identified SNPs that affect gene expression levels in neuronal cells, and are likely to affect expression in the brain, by performing PASSPORT-seq. We believe similar assays should be routinely implemented to screen genetic variants identified by GWAS to identify those that affect gene regulation.

## Supplementary information

Supplementary Materials

Supplementary Figures

Supplementary Tables
