# Supplementary material for: Allele-specific expression and high-throughput reporter assay reveal functional genetic variants associated with alcohol use disorders
Source: Mol Psychiatry. 2019 Sep 2;26(4):1142–51. doi: 10.1038/s41380-019-0508-z (PMC7050407; doi:10.1038/s41380-019-0508-z)
Supplement: Supplementary file 2 — Supplementary Figures [file 41380_2019_508_MOESM2_ESM.pdf]

Supplementary Figures

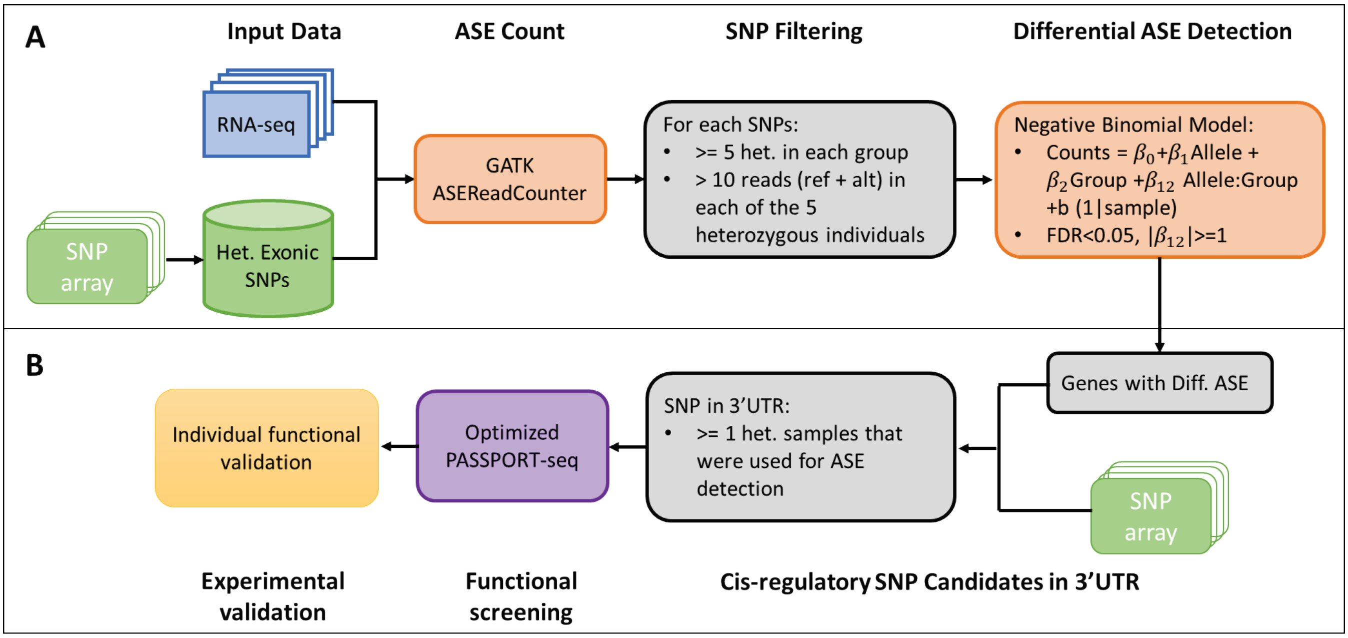

Supplementary Figure 1. Workflow for identifying SNPs in the 3'UTRs of genes with differential allele-specific expression (ASE) in heavy drinkers compared with social/non-drinkers. (A) Heterozygous SNPs are identified in the RNA-sequencing data from brain tissue in social/non-drinkers and heavy drinkers. Differential allele-specific expression is detected using a negative binomial model with random effects. (B) Genes with differential ASE and 3'UTR SNPs in those genes are identified. Passport-seq screening of these SNPs help to identify candidate SNPs for further validation. Het. = heterozygous.

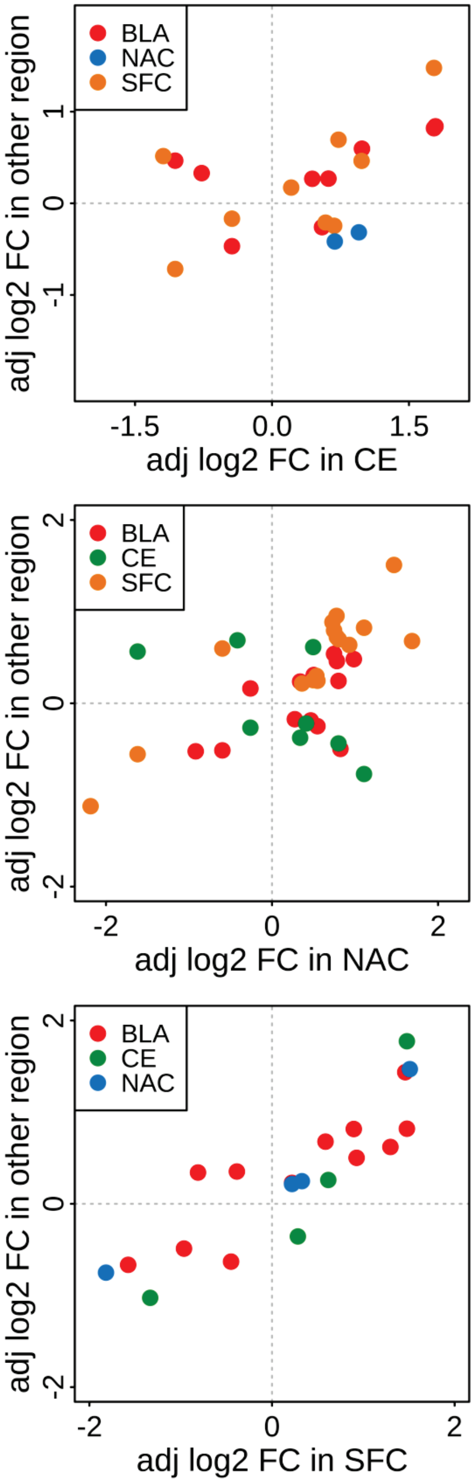

Supplementary Figure 2. Consistency of differential ASE results between brain regions. For each significant (FDR < 0.05) gene in CE that is significant (p < 0.05) in another brain region (BLA, NAC, SFC), the adjusted log2 fold change was plotted. This was repeated for each brain region. Most genes had the same direction of change in each pair of brain regions. BLA = basolateral nucleus of the amygdala, CE = central nucleus of the amygdala, NAC = nucleus accumbens, and SFC = superior frontal cortex.

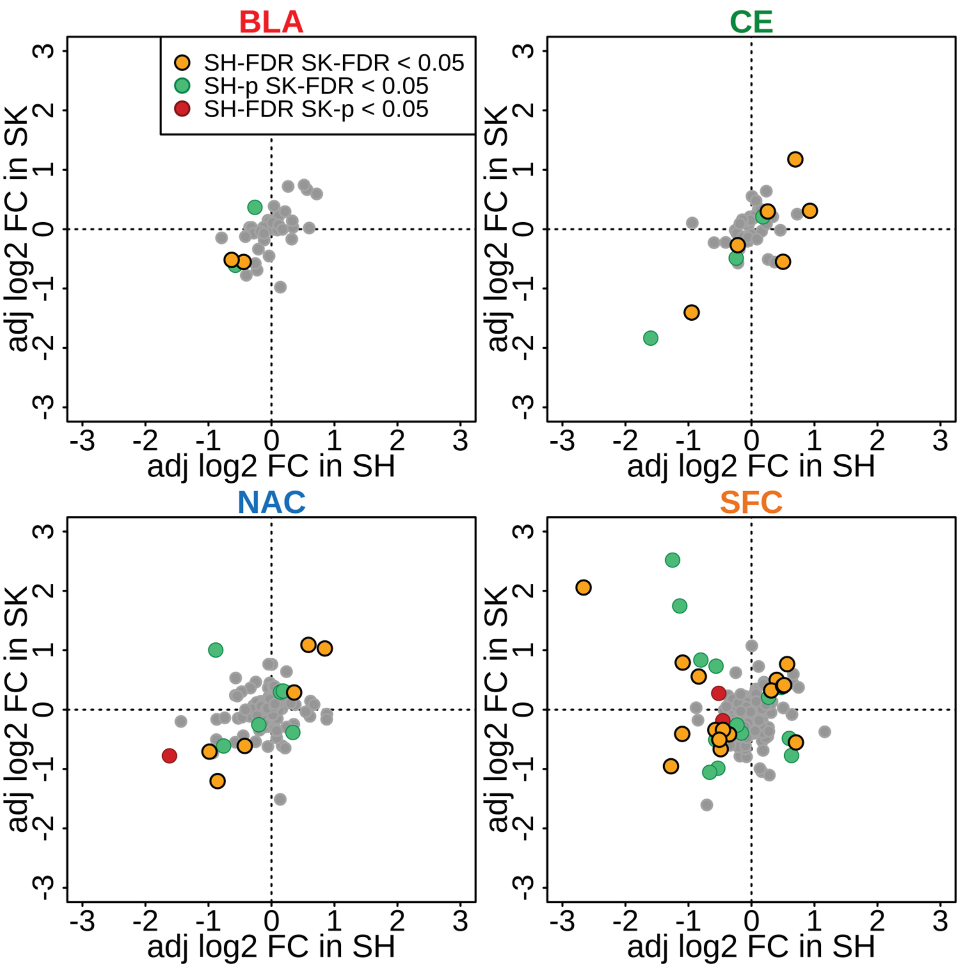

Supplementary Figure 3. Passport-seq results for each brain region (BLA, CE, NAC, and SFC). Significantly differentially expressed genes in SH-SY5Y [SH] and SK-N-BE(2) [SK] cells are color-coded to denote significance.

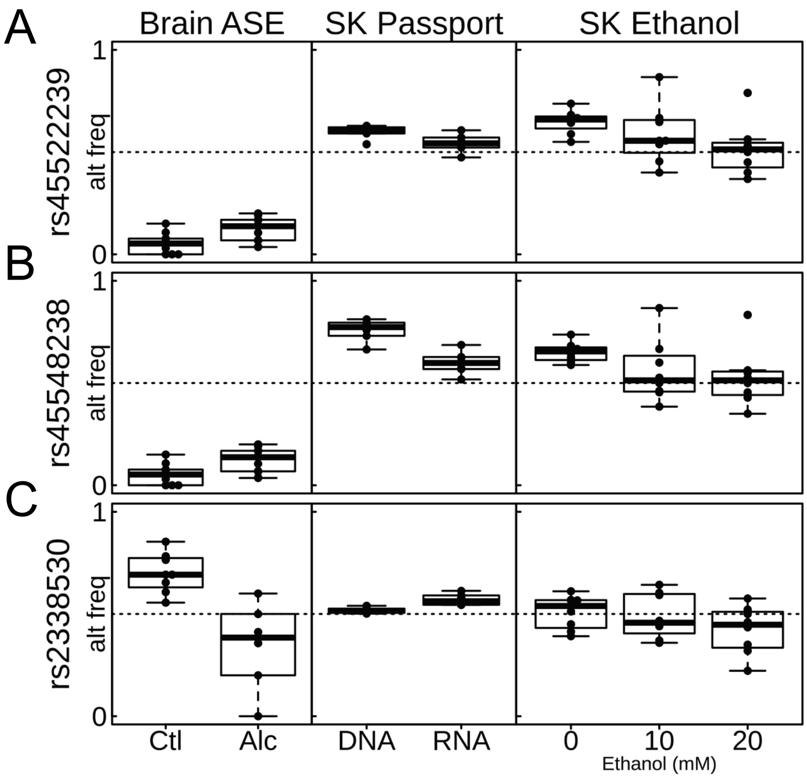

Supplementary Figure 4. ASE, PASSPORT-seq, and ethanol treatment results for additional SNPs with alternative allele expression sensitive to ethanol treatment. Three additional SNPs (rs45522239, rs45548238, rs2338530) that had alternative allele frequency significantly different in PASSPORT-seq results (FDR < 0.05) and significantly different across 0 mM, 10 mM, and 20 mM ethanol treatment dosages in SK-N-BE(2) cells ($p < 0.05$) were identified. The differences in the alternative allele frequency in the brains of heterozygous social/non-drinkers (Ctl) and AUD subjects (Alc) were also shown. SK = SK-N-BE(2) cell.
